# RETRACTED: Choi, H.-S.; Lee, B.-M. A Complex Intervention Integrating Prism Adaptation and Neck Vibration for Unilateral Neglect in Patients of Chronic Stroke: A Randomised Controlled Trial. *Int. J. Environ. Res. Public Health* 2022, *19*, 13479

**DOI:** 10.3390/ijerph23040536

**Published:** 2026-04-21

**Authors:** Hyun-Se Choi, Bo-Min Lee

**Affiliations:** 1 Department of Rehabilitation Medicine, Seoul National University Bundang Hospital, Seongnam 13620, Republic of Korea; 2 Department of Rehabilitation Science, Inje University Graduate School, Gimhae 50834, Republic of Korea

The journal retracts the article titled “A Complex Intervention Integrating Prism Adaptation and Neck Vibration for Unilateral Neglect in Patients of Chronic Stroke: A Randomised Controlled Trial” [1], cited above.

Following the publication, concerns were brought to the attention of the Editorial Office regarding the scientific accuracy of the data presented in the published paper [1].

In accordance with standard journal procedures, the Editorial Office, Research Integrity team and Editorial Board conducted an investigation that confirmed statistical inconsistencies and discrepancies within the data presented in Table 2. Although the authors cooperated during the investigation process, the Editorial Board was unable to verify the validity or integrity of the data. Consequently, the Editorial Board has lost confidence in the reliability of the findings and decided to retract this publication [1], as per MDPI’s retraction policy.

This retraction was approved by the Editor-in-Chief of the journal *International Journal of Environmental Research and Public Health*.

The authors agreed to this retraction.
